# Assessing Delirium Vulnerability in Patients Referred to Consultation‐Liaison Psychiatry Service: A Cross‐Sectional Survey

**DOI:** 10.1002/hsr2.71218

**Published:** 2025-09-09

**Authors:** Faezeh Khorshidian, Farzan Kheirkhah, Sussan Moudi, Davood Hosseini Talari, Ali Bijani, Neda Fathi, Sarah Mohammadi, Minoo Mojarrad, Seyedeh Mahbobeh Mirtabar

**Affiliations:** ^1^ Department of Psychiatry, School of Medicine.Cognitive Neurology, Dementia and Neuropsychiatry Research Center Tehran University of Medical Sciences Tehran Iran; ^2^ Department of Psychiatry, School of Medicine, Health Research Institute Babol University of Medical Sciences Babol Iran; ^3^ Social Determinants of Health Research Center Babol University of Medical Sciences Babol Iran; ^4^ Social Determinants of Health Research Center, Health Research Institute Babol University of Medical Sciences Babol Iran; ^5^ Student Committee Research Babol University of Medical Sciences Babol Iran; ^6^ Department of psychiatry Shahrood University of Medical Sciences and Health Services Shahrood Iran

**Keywords:** consultation, delirium, medical wards, psychiatry

## Abstract

**Background and Aims:**

A large proportion of patients suffering from medical conditions also experience mental health problems, cooperation of both side's professionals, seems to ensure the best way to reduce the existing gap and to create the best diagnostic‐treatment plan, Consultation‐Liaison Psychiatry provides this possibility in a general hospital. Delirium is one of the most important consultations requested by medical professionals for psychiatric services because it is a condition that threatens life and worsens the prognosis of patients. In this study, we conducted a comprehensive investigation of delirium and its related factors among patients in general hospitals.

**Methods:**

This cross‐sectional analytical study was conducted in medical wards of a General Educational hospital in North Iran in 2022, Sampling was done among 864 patients, hospitalized in different medical departments who were requested by their medical specialists for consultation by the psychiatric service, The information collected for this study was obtained based on the recorded data in the psychiatric consultation sheets, All psychiatric diagnoses in this project were based on DSM‐5 diagnostic criteria.

**Results:**

Our analysis revealed a significant increase in the incidence of delirium among patients with advancing age (*p* < 0.001), those with an educational level below diploma (*p* < 0.001), and individuals who are widowed (*p* < 0.001). Additionally, abnormalities in all laboratory tests were significantly correlated with a higher rate of delirium (*p* < 0.001), with the exception of thyroid dysfunction tests and positive urine toxicology results. Regarding disease classification, delirium was most frequently observed in patients with multiple comorbidities, accounting for 55%, followed by those with lung disease at 49.5%. In all types of medical diseases, the incidence of hyperactive and mixed‐state delirium was significantly higher (*p*‐value = 0.009). Additionally, we observed that the majority of patients with delirium had no history of active substance use (81%) or underlying dementia (92.4%).

**Conclusions:**

The results of our study demonstrate that advanced age, lower educational attainment, widowhood, and the presence of multiple comorbidities are significant risk factors for delirium among hospitalized patients. Furthermore, disturbances in laboratory parameters—excluding thyroid dysfunction and positive urine toxicology—are strongly associated with increased delirium risk. Notably, the majority of patients experiencing delirium had no prior history of active substance use or underlying dementia, as evidenced by our findings.

## Summary

- Delirium prevalence was 27.43%, and increased significantly with age; over half of delirium cases were patients above 60 years old.
- Widowed patients had a 3.54 times higher risk of delirium compared to married ones.
- Delirium was most common in ICU patients (64.5%) and those with multiple diseases, lung disease, and complicated diabetes.
- Laboratory abnormalities such as anemia, electrolyte imbalance, and liver/kidney dysfunction were significantly associated with delirium.
- Logistic regression showed that age, widowhood, ICU admission, and organ dysfunction increased delirium risk, while female sex lowered it.

**Abbreviations**
CCU: cardiac care unit
CLP: consultation‐liaison psychiatry
DSM‐5: Diagnostic and Statistical Manual Of Mental Disorders Edition‐5
ICU: intensive care unit

## 1 Background

Individuals with mental disorders are more frequently affected by physical conditions, whereas a large proportion of patients suffering from medical conditions also experience mental health problems [1, 2], Medical‐psychiatric comorbidity is linked to increased length of hospital stay, higher medical costs, and often hospitalization [3, 4].

Consultation‐Liaison Psychiatry (CLP) is a subspecialty of psychiatry that provides assessment and treatment for general hospital patients with mental health comorbidities, It also provides teaching and research activities on mental health comorbidity to medical staff of nonpsychiatric departments of general hospitals [5], cooperation of both sides, between medical and psychiatric professionals seem to ensure the best way to reduce the existing gap, between medical specialties and psychiatrist [6].

The scope, organization, and strategic aspects of CLP in general hospitals/wards have developed worldwide. Previous studies showed that CLP reduces medical complications, length of stay, and number of hospitalizations via early referral for psychiatric consultations and also provides treatment of psychiatric presentation on medical and surgical units and facilitates access to appropriate post‐discharge psychiatric treatment [7, 8, 9, 10]. Although CLP is a relatively new specialty, in the last decade service‐based research in this area has made progress in quantifying CLP activity and providing the best practice of its services, as well as defining the needs and future development of this branch of psychiatry [11, 12, 13]. Delirium is one of the most important disorders due to which, medical disease specialists seek advice from psychiatry services including CLP. Delirium is a syndrome of acute brain failure that is the direct pathophysiologic consequence of an underlying medical condition or toxic exposure [14, 15]. According to DSM‐5 criteria, it is characterized by the acute onset of deficits in attention, awareness, and cognition that fluctuate in severity over time. Delirium represents global brain dysfunction and thus its cognitive impairments are highly variable, including disturbances in several domains, such as memory, orientation, language, visuospatial ability, and perception [16]. Additional features include psychomotor disturbance, altered sleep cycle, and emotional variability. The psychomotor disturbances seen in delirium have been categorized into three phenotypic subtypes: hyperactive, hypoactive, and mixed‐state delirium [16, 17, 18]. Hyperactive delirium is characterized by psychomotor agitation, restlessness, and emotional lability and is sometimes mistaken for primary psychosis, mania, or dementia. Hypoactive delirium is characterized by psychomotor retardation, lethargy, and decreased level of responsiveness and is often missed or misdiagnosed as depression. Mixed delirium presents with alternating features of both [17, 18]. In the recent systematic review study, the most reported symptoms were inattention, disorientation, psychomotor agitation/retardation, hallucination and memory impairment. Notably, psychomotor agitation and hallucinations are not listed in the current Diagnostic and Statistical Manual for Mental Disorders‐5‐Text Revision delirium definition [19]. The incidence of delirium on general medical wards ranges from 11% to 42% and it is as high as 87% among critically ill patients [17]. Although delirium is common, the diagnosis relies on a high index of suspicion, as it often goes undetected or misdiagnosed [14, 15].

There are multiple challenges to establishing the diagnosis of delirium in a timely fashion, including the fluctuating course of symptoms, difficulty conducting cognitive testing during the extremes of psychomotor disturbance, overlooking the hypoactive phenotype, and the need to ascertain baseline cognitive functioning [16, 17]. A thorough clinical evaluation is considered the gold standard for the diagnosis of delirium, as there is no clinical study or biomarker with high sensitivity and specificity. Although EEG studies typically show generalized slowing in delirium, the false negative and false positive rates approach 20%, limiting the utility of this tool [18, 20, 21].

Delirium in medically ill patients is often multifactorial, and while important attention needs to be given to extensive surveillance and monitoring of contributing variables, the role of the psychiatric consultant often includes a special focus on potential neuropsychiatric processes [22, 23, 24, 25]. A broader differential diagnosis can be considered in the context of the patient's presentation at the time of consultation [26].

The risk of delirium is determined by predisposing risk factors (i.e., the background characteristics of patients) and precipitating risk factors (i.e., acute insults, injury, or drugs) [27, 28, 29]. Neuroimaging studies indicate that the risk of delirium might be higher in individuals with greater cerebral atrophy and/or greater white matter disease [30, 31, 32]. Genetic studies have not identified consistent candidate genes associated with delirium risk but these studies are few and underpowered [29]. Precipitating factors for delirium span a wide range of different kinds of causes, including, acute medical illness, trauma, surgery, dehydration, and psychological stress [33, 34, 35]. In addition, drug use withdrawal, and medication changes are associated with delirium. Of note, benzodiazepines, dihydropyridines, antihistamines, and opioids may convey the highest risk of delirium, although insufficiently managed pain may itself be a risk factor; however, the exact relationship between pain medication, pain management, and delirium risk remains unclear [34, 35]. In addition to common precipitating factors, specific healthcare setting‐related factors, such as mechanical ventilation are risk factors for hospital‐acquired delirium. Many of these factors may coexist in different health‐care settings [29, 35].

### 1.1 Importance of the Study and Hypothesis

Despite the importance of consultant liaison psychiatry in identifying and treating psychiatric disorders as quickly as possible in general hospitals, the studies conducted in this field in Iran are still limited and more research is needed, On the other hand, delirium is one of the most important consultations requested by medical professionals to psychiatric services, because it is very influential in the overall prognosis of patients and their lives and identifying delirium as well as its related factors as soon as possible is very important in the course of patients medical conditions. Our hypothesis is that the rate of delirium is higher in medically hospitalized patients. Until now, studies in the field of delirium have been limited, especially considering its *activity state,* So in this study, in addition to a complete investigation of delirium and its related factors, we also paid special attention to its *activity state,* which is the *superiority and novelty* of our study design, compared to previous studies.

## 2 Methods

### 2.1 Study Design

This cross‐sectional analytical study was conducted in medical wards of a General Educational hospital affiliated with the University of Medical Sciences in North Iran in 2022, Sampling was done among patients, hospitalized in different medical departments, who were requested by their medical specialists for consultation and visits by the psychiatric service in 2022, the statistical specialist of research reached the sample size by using the formula. In this study, with a confidence level of 95% and the assumption of *p* = 10% for psychiatric disorders and with *d* = 0.02 and based on the statistical $N=\frac{Z1-\frac{\alpha 2}{2}}{d^{2}}pq$ formula: First, the files of all 1100 inpatients who were consulted by the psychiatric service during 1 year were initially examined, but finally, according to the entry and exit criteria of this study, 864 participants remained in the study. All these patients were subjected to a structured interview based on DSM‐5. The informed consent forms, participants' recruitment materials, and research protocol were investigated and certified by institutional ethics committees. Written informed consent was given by executors of the study to all participants. The forms were based on local regulatory requirements and adhered to the ethical principles of the Declaration of Helsinki with the code of ethics: (IR.MUBABOL.HRI.REC.1402.049). After the project was approved by the research unit, the code of ethics was approved by the National Ethics Unit of the University of Medical Sciences.

### 2.2 The Characteristics of Participants

The study was conducted by a team of psychiatric specialists among 864 patients who were admitted to different medical departments in a hospital in northern Iran, in 2022, for whom psychiatric consultation was requested by their medical specialist. Psychiatric consultations in our study were requested by the treating physicians based on clinical judgment, often due to a variety of neuropsychiatric symptoms such as altered level of consciousness, agitation, confusion, suspiciousness, sleep disturbances, mood changes, or abnormal behaviors observed during hospitalization. The decision was primarily based on the physician's clinical impression.

### 2.3 Eligibility Criteria

Inclusion criteria included: Patients who are over 18 years of age and are hospitalized and have been requested to consult with a psychiatric specialist by their physician.

In this study patients with mental retardation and patients who were not able to respond and cooperate and did not have a companion were excluded.

### 2.4 Study Tools

The information collected for this study was obtained based on the information recorded in the psychiatric consultation sheets of the patients that were asked by the psychiatrists of the research. In this project, in addition to examining delirium and its types (states) and factors related to delirium, the association of delirium with dementia and active substance use in patients was also evaluated. All psychiatric diagnoses in this project were based on DSM‐5 diagnostic criteria, which is the planned fifth edition of the American Psychiatric Association, it is diagnostic and statistical manual of mental disorders that was published in May 2013, although according to the condition of the patients, obtaining history of active substance use and history of underlying dementia was based on the history of patients' companions.

In this study, demographic information, including age, gender, educational level, marital status of the patients was obtained from the information contained in the clinical file of the patients and also the information obtained from the patients themselves and their companions during the history taking, Also, the type of medical disease of the patients was obtained and recorded based on the final diagnosis recorded in their medical hospitalization file(classified as neurological/gastrointestinal/cancer and hematology/neurosurgery/cardiovascular/pulmonary/diabetes and its complications/multiple diseases/others). The type of laboratory disorder of the patients was obtained and recorded based on the paraclinical information recorded in the patient file and also the computer file of the patient's laboratory (divided into Renal/liver/thyroid/electrolytes/complete blood count, Diff/urine toxicology/other tests in this study), the type of medical ward in which the patients were admitted was recorded on the consultation sheets based on the seal related to the ward (divided into internal/surgical/cardiology and CCU/ICU/infectious/neurology departments), after the data were collected, they were subjected to statistical analysis by the statistician of the project.

### 2.5 Statistical Analysis

Data were entered into SPSS software version 22, descriptive statistics were used using mean and standard deviation for quantitative data and frequency and ratio for qualitative data, and *χ* ^2^ tests, *t*‐test, ANOVA, and Logistic regression model were used. Data were analyzed and the level of statistical significance was considered 0.05.

## 3 Results

As mentioned in Table 1, 53% of patients were female and 47% were male. In terms of age, most of the patients (41.6%) were over 60 years old. In terms of marital status, most of them (74.4%) were married and in terms of educational level, most of them (66.7%) had below diploma educational level. It was found that the rate of delirium increased significantly with increasing age of the patients and 50.7% of delirium patients were over 60 years old (*p*‐value < 0.001), Also, people whose spouse had died (*p*‐value < 0.001) and patients with below diploma educational level showed significantly more delirium (*p*‐value < 0.001). In this study, there was no significant relationship between gender and delirium (*p*‐value = 0.399), In general, in our study, the rate of delirium among hospitalized patients was 27.43%.

**Table 1:** Frequency distribution of participants' demographics and their association with delirium.

		Percent	Delirium		*p*‐value delirium
		(%)	No	Yes	
Age	18–25	7.5	63 (96.9)	2 (3.1)	< 0.001
	25–40	19.7	157 (93.5)	11 (6.5)	
	40–60	31.2	225 (84.3)	42 (15.7)	
	> 60	41.6	177 (49.3)	182 (50.7)	
Marital status	married	74.4	486 (76.4)	150 (23.6)	< 0.001
	Single	8.1	64 (94.1)	4 (5.9)	
	Divorced	4.4	35 (94.6)	2 (5.4)	
	Widow	13.1	34 (30.4)	78 (69.6)	
Sex	Female	53.0	320 (71)	131 (29)	0.399
	Male	47.0	295 (73.8)	105 (26.3)	
Level of education	Below Diploma	66.7	362 (64.1)	203 (35.9)	< 0.001
	Diploma to Bachelor	25.6	195 (89.9)	22 (10.2)	
	Higher than Bachelor	7.7	58 (87.9)	8 (12.1)	

As mentioned in Table 2, patients with positive laboratory tests of Anemia (*p*‐value < 0.001) electrolytes disturbance (*p*‐value < 0.001), liver function disturbance (*p*‐value < 0.001) kidney function disturbance (*p*‐value < 0.001) and also other laboratory tests disturbance (*p*‐value < 0.001), had significantly more delirium. On the other hand, most of the patients who developed delirium were negative in terms of urine toxicology tests (*p*‐value < 0.001) and also had normal thyroid function test (*p*‐value = 0.002).

**Table 2:** Correlation between laboratory variables and delirium.

		Delirium		*p*‐value
		No	Yes	
Anemia	No	494 (76)	156 (24)	< 0.001
	Yes	129 (61.4)	81 (38.6)	
TSH disturbance	No	581 (71.4)	233 (28.6)	0.002
	Yes	42 (91.3)	4 (8.7)	
Electrolyte disturbance	No	601 (75)	200 (25)	< 0.001
	Yes	22 (37.3)	37 (62.7)	
Kidney function disturbance	No	588 (76.8)	178 (23.2)	< 0.001
	Yes	35 (37.2)	59 (62.8)	
Liver function disturbance	No	578 (74.4)	199 (25.6)	< 0.001
	Yes	45 (54.2)	38 (45.8)	
Urine toxicology	No	526 (70.2)	223 (29.8)	< 0.001
	Yes	97 (87.4)	14 (12.6)	
Others	No	506 (78.7)	137 (21.3)	< 0.001
	Yes	117 (53.9)	100 (46.1)	

Abbreviation: TSH, thyroid stimulating hormone.

As mentioned in Table 3, the most delirium was related to multiple diseases (patients with more than one disease) with 55%, followed by Lung disease with 49.5% and then with diabetes and its consequences with 43.8%. The relationship between delirium and the type of disease showed statistically significance (*p*‐value < 0.001). In relation to the inpatient departments, the most delirium was observed in the ICU department with 64.5% and then in the infectious department with 50%, which showed a significant relationship between delirium and the inpatient department (*p*‐value < 0.001). There was also a significant relationship between need for re‐counseling and delirium (*p*‐value < 0.001). In this study, most of the patients who had delirium didn't have a history of active substance use (81%) or underlying dementia (92.4%).

**Table 3:** Relationship between re‐counseling and medical conditions, hospital ward, substance use, dementia, and delirium.

		Delirium		*p*‐value
		No	Yes	
Need for re‐consultation	No	546 (87.6)	< 0.001
	Yes	77 (12.4)		53 (22.4)
Type of medical illness	Neurological disease	74 (74.7)	25 (25.3)	< 0.001
	Gastrointestinal disease	97 (80.8)	23 (19.2)	
	Cancer and hematological disease	68 (72.3)	26 (27.7)	
	Diabetes and it's consequences	27 (56.3)	21 (43.8)	
	cardiovascular disease	154 (80.6)	37 (19.4)	
	Lung disease	54 (50.5)	53 (49.5)	
	Neurosurgery disease	36 (90)	4 (10)	
	others	86 (86)	14 (14)	
	Multiple diseases	27 (45)	33 (55)	
Type of inpatient department	Infectious ward	57 (50)	57 (50)	< 0.001
	Internal wards	214 (68.8)	97 (31.2)	
	Neurology ward	70 (77.8)	20 (22.2)	
	Cardiovascular and CCU wards	112 (77.8)	32 (22.2)	
	Surgery wards	142 (96.6)	5 (3.4)	
	ICU	11 (35.5)	20 (64.5)	
Active substance use	No	505 (72.5)	192 (27.5)	0.99
	Yes	118 (72.4)	45 (27.6)	
Dementia	No	573 (72.3)	219 (27.7)	0.888
	Yes	50 (73.5)	18 (26.5)	

Based on our findings, at all ages, most of the patients had hyperactive and mixed state of activity delirium and the relationship between the type of delirium and age was statistically significant (*p*‐value = 0.012), In patients over 60 years old, 53.8% of deliriums were hyperactive type, while in the age group of 25 to 40 years, most of their deliriums (54.5)% were mixed state of activity type. As mentioned in Table 4, among the laboratory disturbances, only the disturbance of thyroid function test and liver function test had statistically significant relationship with the type of delirium and patients with thyroid dysfunction had more mixed type delirium (*p*‐value = 0.039) and patients with liver dysfunction had more hyperactive and mixed state delirium type (*p*‐value = 0.031), It is worth noting that, in general, based on our findings, thyroid function disorder has no significant relationship with delirium, but as recorded in the table below, among delirious patients who had simultaneous thyroid function disorder, between thyroid disorder and type of delirium activity state was found statistically significant relationship (*p*‐value = 0.039).

**Table 4:** Relationship between laboratory variables and type of delirium.

		Delirium			*p*‐value
		Hypoactive	Hyper active	Mix	
Anemia	No	8 (5.1)	73 (46.8)	75 (48.1)	0.241
	Yes	6 (7.4)	45 (55.6)	30 (37)	
TSH disturbance	No	13 (5.6)	118 (50.6)	102 (43.8)	0.039
	Yes	1 (25)	0 (0)	3 (75)	
Electrolyte disturbance	No	13 (6.5)	104 (52)	83 (41.5)	0.143
	Yes	1 (5.9)	14 (49.8)	22 (44.3)	
Kidney function disturbance	No	8 (4.5)	92 (51.7)	78 (43.8)	0.217
	Yes	6 (10.2)	26 (44.1)	27 (45.8)	
Liver function disturbance	No	9 (4.5)	105 (52.8)	85 (42.7)	0.031
	Yes	5 (13.5)	13 (34.2)	20 (52.6)	
Urine toxicology	No	14 (6.3)	110 (49.3)	99 (44.4)	0.825
	Yes	0 (0)	8 (57.1)	6 (42.9)	
Others	No	11 (8)	68 (49.6)	58 (42.3)	0.276
	Yes	3 (5.9)	50 (49.8)	47 (44.3)	

As mentioned in Table 5, in all medical diseases, the rate of hyperactive state delirium and mixed state delirium were statistically significantly higher than hypoactive delirium (*p*‐value = 0.009). The highest rate of hyperactive delirium was seen in patients hospitalized in the infectious department with 66.7% of deliriums, followed by patients hospitalized in ICU with 60% of deliriums. About mixed state delirium, the highest rate of mixed state delirium was 55%, which was seen in the cardiology and CCU departments.

**Table 5:** The relationship between re‐counseling, type of medical disease, type of inpatient department, substance use disorder, dementia variables with the type of delirium.

		Delirium			*p*‐value
		Hypoactive	Hyper active	Mix	
Need for re‐consultation	No	11 (6)	94 (51.1)	79 (42.9)	0.719
	Yes	3 (5.7)	24 (45.3)	26 (49.1)	
Type of medical illness	Neurological disease	0 (0)	11 (44)	14 (56)	0.009
	Gastrointestinal disease	4 (17.4)	6 (26.1)	13 (56.5)	
	Cancer and hematological disease	4 (15.4)	11 (42.3)	11 (42.3)	
	Diabetes and its consequences	1 (4.8)	15 (71.4)	5 (23.8)	
	Cardiovascular disease	1 (2.7)	20 (54.1)	16 (43.2)	
	Lung disease	0 (0)	34 (64.2)	19 (35.8)	
	Neurosurgery disease	1 (25)	1 (25)	2 (50)	
	others	2 (14.3)	6 (42.9)	6 (42.6)	
	Multiple diseases	1 (3)	14 (42.4)	18 (54.5)	
Type of inpatient department	Infectious ward	0 (0)	38 (66.7)	19 (33.3)	0.01
	Internal wards	11 (11.3)	40 (41.2)	46 (47.4)	
	Neurology ward	0 (0)	10 (50)	10 (50)	
	Cardiovascular and CCU wards	0 (0)	13 (40.6)	19 (59.4)	
	Surgery wards	1 (20)	2 (40)	2 (40)	
	ICU	1 (5)	12 (60)	7 (35)	
Active substance use	No	12 (85.7)	90 (76.3)	90 (85.7)	0.18
	Yes	2 (14.3)	28 (23.7)	15 (14.3)	
Dementia	No	13 (92.9)	111 (94.1)	95 (90.5)	0.599
	Yes	1 (7.1)	7 (5.9)	10 (9.5)	

In our research, no statistically significant relationship was found between active substance use and the type of delirium (*p*‐value = 0.18), as well as between the presence of underlying dementia and the type of delirium (*p*‐value = 0.599).

As shown in Table 6, the results of multiple logistic regression showed that the chance of delirium increases significantly with increasing age. In terms of marital status, the chance of delirium increased by 3.54 times in people whose spouse had died. In terms of gender, the chance of women getting delirium decreased by 40%. In all departments, the chance of developing delirium was lower than infectious department, except for the ICU department, where the chance of developing delirium is 1.7 times higher than infectious department.

**Table 6:** Results of multiple logistic regression to predict delirium.

	*B*	SE	Wald	df	*p*‐value	OR	95% CI for OR	
							Lower	Upper
Age (18–25)			43.108	3	0.000			
25–40	1.620	0.928	3.051	1	0.081	5.055	0.821	31.142
40–60	2.494	0.975	6.542	1	0.011	12.105	1.791	81.812
> 60	3.715	0.973	14.567	1	0.000	41.053	6.093	276.601
Marital status (married)			20.749	3	0.000			
Single	0.638	0.739	0.746	1	0.388	1.893	0.445	8.062
Divorced	−0.470	0.795	0.349	1	0.555	0.625	0.131	2.971
Widow	1.265	0.285	19.676	1	0.000	3.544	2.026	6.200
Sex (male/female)	−0.493	0.204	5.829	1	0.016	0.611	0.409	0.911
(Department) infectious ward			41.163	5	0.000			
Internal wards	−0.778	0.296	6.918	1	0.009	0.460	0.257	0.820
Neurology ward	−0.977	0.391	6.255	1	0.012	0.377	0.175	0.810
Cardiovascular and CCU wards	−1.469	0.357	16.900	1	0.000	0.230	0.114	0.464
Surgery wards	−2.716	0.537	25.529	1	0.000	0.066	0.023	0.190
ICU ward	0.534	0.524	1.041	1	0.307	1.706	0.611	4.761
(Laboratory) Electrolyte disturbance (yes/no)	1.165	0.339	11.806	1	0.001	3.207	1.650	6.235
Kidney disturbance (yes/no)	0.958	0.290	10.916	1	0.001	2.607	1.477	4.603
Liver disturbance (yes/no)	0.925	0.310	8.917	1	0.003	2.522	1.374	4.628
Others (yes/no)	0.574	0.226	6.464	1	0.011	1.776	1.141	2.765

Among the laboratory tests, disturbances in liver and kidney tests, electrolyte disturbance and other tests disturbance, compared to CBC, diff (Complete Blood Count) disturbance, thyroid disturbance and urinary toxicology disturbance, had a higher chance of delirium.

## 4 Discussion

Based on our research, 27.43% of hospitalized patients have delirium. Our findings showed that increasing age, being a widow, hospitalization in the ICU and infectious departments, kidney and liver dysfunction, and electrolyte disturbances create the highest risk for delirium. Most delirium is related to multiple diseases (patients with more than one disease) at 55%, followed by Lung disease at 49.5%. In all types of medical diseases, the rate of hyperactive and mixed‐state delirium is statistically significantly higher (*p*‐value = 0.009). The highest rate of hyperactive delirium is related to diabetes and its complications, in which 71.4% of delirium cases are hyperactive, followed by lung diseases with 64.2%. On the other hand, the highest rate of mixed‐state delirium is seen in gastrointestinal diseases with 56.5%. The need for re‐consultation showed a statistically significant relationship with delirium (*p*‐value < 0.001), but it doesn't have a significant relationship with the type of delirium, and the need for re‐consultation is similar in all states. Based on our research, there is no statistically significant relationship between active substance use and delirium (*p*‐value = 0.99), as well as between the presence of underlying dementia and delirium (*p*‐value = 0.888), and most patients with delirium don't have a history of substance abuse(81%) or underlying dementia(92.4%).

Our study is consistent with that described in the literature by Fuchs et al. [36], who points out that the prevalence of delirium in patients older than 65 years is within the range of 11%–50% during their hospitalization. Despite the high prevalence, it is often underdiagnosed or misdiagnosed in up to 70% of cases, in our study, we found that the rate of delirium increased significantly with increasing age of the patients and 50.7% of delirium patients were over 60 years old as mentioned in Table 1. Nguyen Ngoc Tran et al. [37], conducted a study with a convenience sample of nonsurgical patients admitted to emergency rooms and ICU, in their research. Delirium was found in a total of 106 (63.1%) patients. The factors significantly affecting its development were acute or chronic kidney failure, respiratory disorders, malnutrition, hyponatremia, and hypernatremia, In general, the rate of delirium in our study was 27.43%, which was inconsistent with their study, this difference could be due to the difference in the inpatient departments examined in the two studies, our study was conducted on patients in all inpatient departments of the hospital, in while their study was only in ICU and emergency department. On the other hand, in our study, consistent with their study, electrolyte disorder and kidney and lung disease were identified as risk factors for delirium with *p*‐value < 0.001 as mentioned in Table 2. We found that the need for re‐consultation has a statistically significant relationship with delirium (*p*‐value < 0.001), but it hasn't a significant relationship with the type of delirium, and the need for re‐consultation is similar in all states as mentioned in Tables 3 and 5, this is consistent with what was described by Barra et al. [38], that patients with a diagnosis of delirium require at least one more visit by CLP unit psychiatrists than other psychiatric diagnoses. Re‐consultation could be related to the greater clinical complexity of delirium, worsening physical condition, and delay in initial referral to the CLP unit. This higher use of hospital services is closely related to the increase in health costs.

Elie et al. [39], reported in their study, that the prevalence of delirium was 20.8%. In their research, between groups, ≥ 80 vs. 18–80 years, the very old were more commonly admitted as emergencies and developed delirium more frequently, but inconsistently with our study, they report that the number of medical diagnoses has no significant role in the precipitation of the delirium.

Golparvaran, conducted a study among 300 elderly hospitalized in the emergency and internal departments of Firoozabadi and Rasoul Akram hospitals in Tehran, Iran, in their study, 43% of patients had delirium, the patients' delirium status had a significant relationship with the history of cognitive impairment (*r* = 0.350, *p* = 0.002) inconsistent with our research because, in our study, most patients with delirium did not have a history of underlying dementia, this difference can be caused by the difference in the group of people under investigation (they only included elderly patients with mean age 70.91 ± 8.77 years), and older patients have a higher risk of developing dementia, but in our research, we examined patients over the age of 18 hospitalized in different medical departments.

Based on the findings of Jo Ellen et al. [40], neuroimaging studies indicate that the risk of delirium might be higher in individuals with greater cerebral atrophy and/or greater white matter disease, In our study, based on collateral supplementary history, most of the patients were at the level of cognitive functions where they lived independently and even performed instrumental activities before hospitalization, Of course, we did not use neuroimaging evaluations in our study, and this could be the reason for the difference in the results of the two studies.

Sampson et al. [35], reported benzodiazepines, dihydropyridines (l‐type calcium channel blockers typically used in the treatment of hypertension), antihistamines, and opioids may convey the highest risk of delirium, although insufficiently managed pain may itself be a risk factor, however, the exact relationship between pain medication, pain management and delirium risk remains unclear, In our study, most of the patients did not have a positive history of active substance use, and in those patients who had a positive urine toxicology, no statistically significant correlation with delirium was found. One possibility is that according to previous articles [35], pain can be one of the precipitating factors of delirium, Our examined patients also often received opioid drugs in the hospital to control pain, which could lead to the fact that even their delirium will decrease with the reduction of pain.

In our research, we tried to have a comprehensive approach to delirium and its risk factors and we also paid special attention to its activity state, this was the superiority and priority of our study compared to previous studies, and the findings of our project can help in the faster identification and attention of delirium patients and their underlying risk factors, with special attention to their activity level and state, and help in the course of the underlying disease and improve their prognosis. One limitation of this study is related to the method of patient selection. Since only patients for whom psychiatric consultation was requested by the treating physician were included, cases of hypoactive delirium, which often present with subtle symptoms and may be overlooked in routine medical care, might have been underrepresented. This referral bias could have led to underdiagnosis of hypoactive delirium in our sample. Future studies using systematic screening tools for delirium among all hospitalized patients, regardless of consultation requests, would help to address this limitation. However, the impossibility of obtaining a reliable history of substance use and underlying dementia directly from the patients themselves, due to their delirium, required us to rely on information provided by the patients' companions. This reliance on collateral history represents another limitation of our study, as such information may not always be accurate or complete. Therefore, we emphasize the inability to infer causation from these findings and acknowledge the potential biases introduced by self‐reported and second‐hand data.

### 4.1 Suggestions for Future Studies

We suggest that in addition to clinical interviews, a questionnaire be used to reinforce the data. We also suggest designing similar studies by evaluating patients for a longer duration period, until after the complete remission of delirium, it is possible to obtain a detailed history of the mentioned issues from the patients themselves, which can support the robustness of the data report. In addition, with long‐term follow‐up of patients, a more detailed investigation of long‐term cognitive sequels of delirium can be achieved.

## 5 Conclusion

Our results showed a significant risk of low educational level, increasing age, being a widow, and the presence of multiple medical diseases as risk factors for delirium in hospitalized patients, as well as laboratory disturbances related to risk, except for thyroid dysfunction and positive urine toxicology. Generally, the rate of hyperactive delirium and mixed activity in hospitalized patients is higher than the rate of hypoactive delirium. According to our research, most patients with delirium do not have a history of active substance use or underlying dementia.

## Author Contributions

**Faezeh Khorshidian:** data curation, visualization, supervision, writing – review and editing, writing – original draft, project administration, investigation, conceptualization, validation, resources. **Farzan Kheirkhah:** visualization, supervision, project administration, data curation, conceptualization. **Sussan Moudi:** visualization, supervision, project administration, data curation, conceptualization. **Davood Hosseini Talari:** visualization, supervision, project administration, data curation, conceptualization. **Ali Bijani:** methodology, formal analysis, conceptualization, validation, software. **Neda Fathi:** data curation. **Sarah Mohammadi:** investigation, conceptualization. **Minoo Mojarrad:** data curation. **Seyedeh Mahbobeh Mirtabar:** data curation.

## Ethics Statement

The initial proposal of this study was approved by the Ethics Committee of Babol University of Medical Sciences, Babol, Iran, by the code of ethics: (IR.MUBABOL.HRI.REC.1402.049).

## Consent

Before the study, the objectives of the study were fully explained to the volunteers and informed consent was obtained from all participants of the study. All participants and all researchers and authors of the article consent to publish this article.

## Conflicts of Interest

The authors declare no conflicts of interest.

## Transparency Statement

The lead author Faezeh Khorshidian affirms that this manuscript is an honest, accurate, and transparent account of the study being reported; that no important aspects of the study have been omitted; and that any discrepancies from the study as planned (and, if relevant, registered) have been explained.

## Data Availability

The data that support the findings of this study are available from the corresponding author upon reasonable request.

## References

[1] A. Steffen , J. Nübel , F. Jacobi , J. Bätzing , and J. Holstiege , “Mental and Somatic Comorbidity of Depression: A Comprehensive Cross‐Sectional Analysis of 202 Diagnosis Groups Using German Nationwide Ambulatory Claims Data,” BMC Psychiatry 20 (2020): 142. 32228541 10.1186/s12888-020-02546-8 PMC7106695

[2] L. S. C. Woon , H. B. Sidi , A. Ravindran , et al., “Depression, Anxiety, and Associated Factors in Patients With Diabetes: Evidence From the Anxiety, Depression, and Personality Traits in Diabetes Mellitus (ADAPT‐DM) Study,” BMC Psychiatry 20 (2020): 227. 32397976 10.1186/s12888-020-02615-y PMC7218550

[3] L. Jansen , M. van Schijndel , J. van Waarde , and J. van Busschbach , “Health‐ Economic Outcomes in Hospital Patients With Medical‐Psychiatric Comorbidity: A Systematic Review and Meta‐Analysis,” PLoS One 13 (2018): e0194029. 29534097 10.1371/journal.pone.0194029 PMC5849295

[4] Z. Kovacs , M. Asztalos , S. Grøntved , and R. E. Nielsen , “Quality Assessment of a Consultation‐Liaison Psychiatry Service,” BMC Psychiatry 21 (2021): 281. 34074240 10.1186/s12888-021-03281-4 PMC8167950

[5] A. House , R. West , C. Smith , S. Tubeuf , E. Guthrie , and P. Trigwell , “The Effect of a Hospital Liaison Psychiatry Service on Inpatient Lengths of Stay: Interrupted Time Series Analysis Using Routinely Collected NHS Hospital Episode Statistics,” BMC Psychiatry 20 (2020): 27. 31992254 10.1186/s12888-020-2441-8 PMC6988241

[6] B. Stein , M. M. Müller , L. K. Meyer , and W. Söllner , “Psychiatric and Psychosomatic Consultation‐Liaison Services in General Hospitals: A Systematic Review and Meta‐Analysis of Effects on Symptoms of Depression and Anxiety,” Psychotherapy and Psychosomatics 89 (2020): 6–16. 31639791 10.1159/000503177

[7] D. Shinjo , H. Tachimori , K. Maruyama‐Sakurai , K. Fujimori , N. Inoue , and K. Fushimi , “Consultation‐Liaison Psychiatry in Japan: A Nationwide Retrospective Observational Study,” BMC Psychiatry 21 (2021): 235. 33952238 10.1186/s12888-021-03241-y PMC8097923

[8] *OECD: Health at a Glance 2019: OECD Indicators*. (OECD Publishing, 2019).

[9] S. Davies , T. Iwajomo , C. de Oliveira , J. Versloot , R. Reid , and P. Kurdyak , “The Impact of Psychiatric and Medical Comorbidity on the Risk of Mortality: A Population‐Based Analysis,” Psychological Medicine 51 (2019): 1–9. 10.1017/S003329171900326X 31775914

[10] “Clinical Guide for Consultation‐Liaison Psychiatry Team.” The Japanese Society of General Hospital Psychiatry: Liaison Multidisciplinary Committee. Japanese, Seiwa‐Shoten, published 2019.

[11] C. Zhou , T. Rajaratnam , S. Abbey , R. Hawa , K. Sheehan , and S. Sockalingam , “Measuring Quality of Care in Consultation Liaison Psychiatry: Outcomes From Two Canadian Hospitals,” General Hospital Psychiatry 54 (2018): 54–56. 29428224 10.1016/j.genhosppsych.2018.01.010

[12] A. Walker , J. R. Barrett , W. Lee , et al., “Organisation and Delivery of Liaison Psychiatry Services in General Hospitals in England: Results of a National Survey,” BMJ Open 8 (2018): e023091. 10.1136/bmjopen-2018-023091 PMC6120655 30173160

[13] A. M. Doherty , R. Plunkett , K. McEvoy , et al., “Consultation‐Liaison Psychiatry Services in Ireland: A National Cross‐Sectional Study,” Frontiers in Psychiatry 12 (2021): 748224. 34912252 10.3389/fpsyt.2021.748224 PMC8666631

[14] R. P. Thom , N. C. Levy‐Carrick , M. Bui , and D. Silbersweig , “Delirium,” American Journal of Psychiatry 176 (2019): 785–793. 31569986 10.1176/appi.ajp.2018.18070893

[15] C. C. H. Chen , H. C. Li , J. T. Liang , et al., “Effect of a Modified Hospital Elder Life Program on Delirium and Length of Hospital Stay in Patients Un‐ Dergoing Abdominal Surgery: A Cluster Randomized Clinical Trial,” JAMA Surgery 152 (2017): 827–834. 28538964 10.1001/jamasurg.2017.1083 PMC5710459

[16] *American Psychiatric Association: Diagnostic and Statistical Manual of Mental Disorders: DSM‐5*, ed 5. (American Psychiatric Association, 2013).

[17] J. R. Maldonado , “Acute Brain Failure,” Critical Care Clinics 33 (2017): 461–519. 28601132 10.1016/j.ccc.2017.03.013

[18] M. R. Agar , P. G. Lawlor , S. Quinn , et al., “Efficacy of Oral Risperidone, Haloperidol, or Placebo for Symptoms of Delirium Among Patients in Palliative Care,” JAMA Internal Medicine 177 (2017): 34. 27918778 10.1001/jamainternmed.2016.7491

[19] E. M. L. Bowman , A. M. Sweeney , D. F. McAuley , et al., “Assessment and Report of Individual Symptoms in Studies of Delirium in Postoperative Populations: A Systematic Review,” Age and Ageing 53 (2024): afae077. 38640126 10.1093/ageing/afae077 PMC11028403

[20] S. T. Williams , J. K. Dhesi , and J. S. L. Partridge , “Distress in Delirium: Causes, Assessment and Management,” European Geriatric Medicine 11 (2020): 63–70. 32297237 10.1007/s41999-019-00276-z

[21] A. J. C. Slooter , W. M. Otte , J. W. Devlin , et al., “Updated Nomenclature of Delirium and Acute Encephalopathy: Statement of Ten Societies,” Intensive Care Medicine 46 (2020): 1020–1022. 32055887 10.1007/s00134-019-05907-4 PMC7210231

[22] P. Casey , W. Cross , M. W. S. Mart , C. Baldwin , K. Riddell , and P. Dārziņš , “Hospital Discharge Data Under‐Reports Delirium Occurrence: Results From a Point Prevalence Survey of Delirium in a Major Australian Health Service,” Internal Medicine Journal 49 (2019): 338–344. 30091294 10.1111/imj.14066

[23] M. A. Oldham and R. G. Holloway , “Delirium Disorder: Integrating Delirium and Acute Encephalopathy,” Neurology 95 (2020): 173–178. 32518149 10.1212/WNL.0000000000009949

[24] A. Teodorczuk and A. MacLullich , “New Waves of Delirium Understanding,” International Journal of Geriatric Psychiatry 33 (2018): 1417–1419. 29314268 10.1002/gps.4848

[25] A. S. Khachaturian , K. M. Hayden , J. W. Devlin , et al., “International Drive to Illuminate Delirium: A Developing Public Health Blueprint for Action,” Alzheimer's & Dementia 16 (2020): 711–725. 10.1002/alz.12075 32212231

[26] K. Gibb , A. Seeley , T. Quinn , et al., “The Consistent Burden in Published Estimates of Delirium Occurrence in Medical Inpatients Over Four Decades: A Systematic Review and Meta‐Analysis Study,” Age and Ageing 49 (2020): 352–360. 32239173 10.1093/ageing/afaa040 PMC7187871

[27] J. Watt , A. C. Tricco , C. Talbot‐Hamon , et al., “Identifying Older Adults at Risk of Delirium Following Elective Surgery: A Systematic Review and Meta‐Analysis,” Journal of General Internal Medicine 33 (2018): 500–509. 29374358 10.1007/s11606-017-4204-x PMC5880753

[28] D. Greaves , P. J. Psaltis , T. J. Ross , et al., “Cognitive Outcomes Following Coronary Artery Bypass Grafting: A Systematic Review and Meta‐Analysis of 91,829 Patients,” International Journal of Cardiology 289 (2019): 43–49. 31078353 10.1016/j.ijcard.2019.04.065 PMC6548308

[29] J. E. Wilson , M. G. Mart , C. Cunnigham , et al., “Delirium,” Nature Reviews Disease Primers 6 (2020): 90, 10.1038/s41572-020-00223-4. PMC9012267 33184265

[30] A. Velayati , M. Vahdat Shariatpanahi , E. Shahbazi , and Z. Vahdat Shariatpanahi , “Association Between Preoperative Nutritional Status and Postoperative Delirium in Individuals With Coronary Artery Bypass Graft Surgery: A Prospective Cohort Study,” Nutrition 66 (2019): 227–232. 31357095 10.1016/j.nut.2019.06.006

[31] I. Persico , M. Cesari , A. Morandi , et al., “Frailty and Delirium in Older Adults:A Systematic Review and Meta‐Analysis of the Literature,” Journal of the American Geriatrics Society 66 (2018): 2022–2030. 30238970 10.1111/jgs.15503

[32] A. Nitchingham , V. Kumar , S. Shenkin , K. J. Ferguson , and G. A. Caplan , “A Systematic Review of Neuroimaging in Delirium: Predictors, Correlates and Consequences,” International Journal of Geriatric Psychiatry 33 (2018): 1458–1478. 28574155 10.1002/gps.4724

[33] T. McCoy , K. Jr Hart , A. Pellegrini , and R. Perlis , “Genome‐Wide Association Identifies a Novel Locus for Delirium Risk,” Neurobiology of Aging 160 (2018): e9–160.e114. 10.1016/j.neurobiolaging.2018.03.008 PMC5993590 29631748

[34] J. Cirbus , A. M. J. MacLullich , C. Noel , E. W. Ely , R. Chandrasekhar , and J. H. Han , “Delirium Etiology Subtypes and Their Effect on Six‐Month Function and Cognition in Older Emergency Department Patients,” International Psychogeriatrics 31 (2019): 267–276. 30021661 10.1017/S1041610218000777

[35] E. L. Sampson , E. West , and T. Fischer , “Pain and Delirium: Mechanisms, Assessment, and Management,” European Geriatric Medicine 11 (2020): 45–52. 32297242 10.1007/s41999-019-00281-2

[36] S. Fuchs , L. Bode , J. Ernst , J. Marquetand , R. von Känel , and S. Böttger , “Delirium in Elderly Patients: Prospective Prevalence Across Hospital Services,” General Hospital Psychiatry 67 (2020): 19–25. 32911278 10.1016/j.genhosppsych.2020.08.010

[37] N. N. Tran , T. P. N. Hoang , and T. K. T. Ho , “Diagnosis and Risk Factors for Delirium in Elderly Patients in the Emergency Rooms and Intensive Care Unit of The National Geriatric Hospital Emergency Department: A Cross‐Sectional Observational Study,” International Journal of General Medicine 14 (2021): 6505–6515. 34675618 10.2147/IJGM.S325365 PMC8518479

[38] B. J. Barra , M. Barahona , L. F. Varela , et al., “A Cross‐Sectional, Retrospective, and Comparative Study Between Delirium and Non‐Delirium Psychiatric Disorders in a Psychogeriatric Inpatient Population Referred to Consultation‐Liaison Psychiatry Unit,” Medicina 59 (2023): 693. 37109651 10.3390/medicina59040693 PMC10141533

[39] J. Marquetand , L. Bode , and S. Fuchs , et al., “Risk Factors for Delirium Are Different in the Very Old: A Comparative One‐Year Prospective Cohort Study of 5,831 Patients,” Front Psychiatry, Aging Psychiatry 12 (2021): 655087, 10.3389/fpsyt.2021.655087. PMC8144286 34045981

[40] J. E. Wilson , M. G. Mart , C. Cunningham , et al., “Delirium,” Nature Reviews Disease Primers 6, no. 1 (2020): 90, 10.1038/s41572-020-00223-4. PMC9012267 33184265

